# Serum PD-1 levels measured by ELISA using Nivolumab increased in advanced RCC patients: novel approach to develop companion diagnostics for antibody therapy

**DOI:** 10.1007/s00432-018-2806-2

**Published:** 2018-12-05

**Authors:** Kosuke Mizutani, Kengo Horie, Taku Kato, Keita Nakane, Kyojiro Kawakami, Yasunori Fujita, Masafumi Ito

**Affiliations:** 1 Department of Urology, Gifu University Graduate School of Medicine, 1-1 Yanagido, Gifu, Gifu 501-1194 Japan; 2 Research Team for Mechanism of Aging, Tokyo Metropolitan Institute of Gerontology, 35-2 Sakae-cho, Itabashi-ku, Tokyo, 173-0015 Japan

**Keywords:** Companion diagnostics, Nivolumab, Programmed cell death-1, Programmed cell death ligand-1, Renal cell carcinoma

Dear Editor,

Antibody therapy targeting specific molecules has been successfully used to treat various diseases including RCC, but some patients do not respond to the therapy (Motzer et al. 2015). There is an urgent need to develop companion diagnostics to select patients that well respond to antibody therapy. PD-L1 on cancer cells suppresses immune response by binding to PD-1 on activated T cells. Antibodies targeting either PD-1 or PD-L1 block their interaction and restore immune response against cancer cells (Rijnders et al. 2017). PD-L1 expression evaluated by immunohistochemistry was used as a predictive biomarker in several studies (Gibney et al. 2016; Rijnders et al. 2017). However, its predictive role in the effect of human anti-PD-1 monoclonal antibody, Nivolumab, in RCC remains unclear (Rijnders et al. 2017). There are also few studies that have examined the predictive value of serum PD-1 levels in RCC patients (Zhu and Lang 2017).

Antibody-based assays to determine the level of target molecules in serum and tissues are highly dependent on the epitope recognized by the antibody. We hypothesized that the use of therapeutic antibody, Nivolumab, rather than other PD-1 antibodies as a capture antibody in ELISA would logically give more reliable and clinically relevant results regarding the serum level of soluble form of PD-1 (Fig. 1a).

**Fig. 1:**
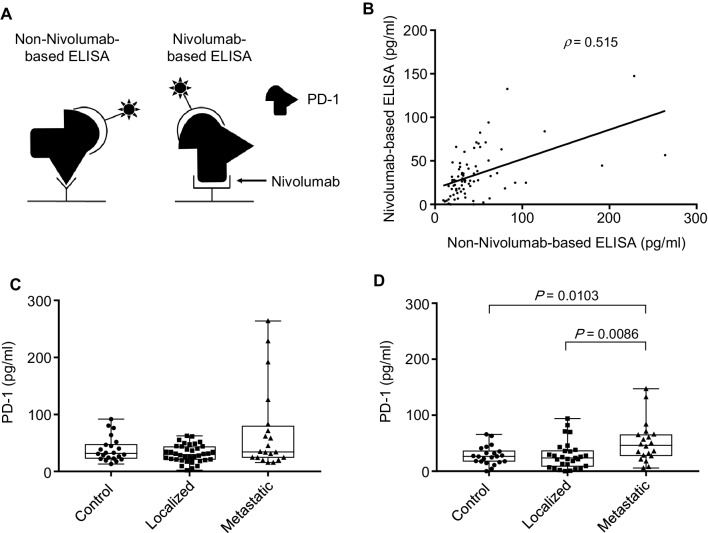
Measurement of serum PD-1 levels in RCC patients. **a** The schema to measure serum PD-1 levels using non-Nivolumab-based ELISA and Nivolumab-based ELISA. **b** The correlation of serum PD-1 levels of all participants between non-Nivolumab-based ELISA and Nivolumab-based ELISA (*ρ* = 0.515, Spearman’s correlation coefficients). **c** The serum PD-1 levels measured using non-Nivolumab-based ELISA in controls, localized patients and metastatic patients (*P* = 0.165, non-parametric one-way ANOVA, Kruskal–Wallis test). **d** The serum PD-1 levels measured using Nivolumab-based ELISA in controls, localized patients and metastatic patients (*P* = 0.013, non-parametric one-way ANOVA, Kruskal–Wallis test). There was a statistical difference between metastatic patients and the other two groups (*P* = 0.0103 metastatic vs. control, *P* = 0.0086 metastatic vs. localized, Mann–Whitney *U* test)

## Methods

After approval by the Review Board of Gifu University, 22 healthy individuals, 29 localized RCC patients and 20 RCC patients with metastasis found at initial diagnosis or at recurrence were studied. The serum PD-1 level was determined using human PD-1 ELISA kit (Thermo Fisher Scientific, MA, USA). For Nivolumab-based ELISA, the capture antibody recognizing PD-1 supplied in the ELISA kit was replaced with Nivolumab kindly provided by Ono pharmaceutical (Osaka, Japan).

## Results

Moderate correlation was observed in the serum PD-1 levels of all participants between non-Nivolumab-based ELISA and Nivolumab-based ELISA (Fig. 1b). Non-Nivolumab-based ELISA showed no statistical difference in the PD-1 levels among the three groups in part due to higher background (Fig. 1c). In Nivolumab-based ELISA, medians of the PD-1 levels were 26.4, 23.7 and 46.4 pg/ml in controls, localized patients and metastatic patients, respectively, and statistically different among the three groups. The serum PD-1 levels in metastatic patients were statistically higher compared to the other two groups (Fig. 1d).

Of the 29 localized RCC, 3 relapsed on 354, 533 and 575 days after surgery (mean observation period was 487.4 days, 95% CI: 360.9–614.0) and their serum PD-1 levels prior to surgery were relatively high (94.2, 36.1 and 71.2 pg/ml, respectively). Among 20 metastatic patients, 6 were treated with Nivolumab. Their serum PD-1 levels just before treatment were 28.3, 34.7, 44.5, 79.1, 111.6 and 147.4 pg/ml, and the first three patients with relatively lower PD-1 levels responded to the treatment.

## Discussion

PD-1 is expressed on tumor-infiltrating lymphocytes and its soluble form is measured in several cancers (Ni et al. 2015; Rijnders et al. 2017). We demonstrated that serum PD-1 levels increased in advanced RCC patients, when measured by Nivolumab-based ELISA. High serum PD-1 levels in metastatic RCC patients may be a consequence of increased infiltrating lymphocytes due to expanded tumor burden. The limitation of this study is the lack of enough data for evaluating the predictive role due to small number of patients. Nevertheless, our pilot study has provided the basis for future large-scale studies to explore the value of PD-1 levels in predicting the response to Nivolumab. More importantly, our results suggest the possibility that serum levels of target molecules measured by therapeutic antibody-based ELISA could be a predictive marker for other antibody therapies as well.

In conclusion, we propose the importance of measuring the serum levels of target molecules with the exact binding epitope using therapeutic antibody, thus providing a novel approach to develop companion diagnostics for antibody therapy, which may well be applicable to other diseases.

## Abbreviations

PD-1: Programmed cell death-1
PD-L1: Programmed cell death ligand-1
RCC: Renal cell carcinoma
